# Protocol for fibrin-based endothelial-cardiomyocyte co-culture in a microfluidic device

**DOI:** 10.1016/j.xpro.2026.104562

**Published:** 2026-05-12

**Authors:** Talitha C.F. Spanjersberg, Aram Klaassen, Richard Wubbolts, Christian G.M. van Dijk, Jolanda van der Velden, Rogier J.A. Veltrop, Pim van der Harst, Magdalena Harakalova, Frank G. van Steenbeek

**Affiliations:** 1 Regenerative Medicine Center Utrecht (RMCU), University Medical Center Utrecht, Utrecht University, Utrecht, the Netherlands; 2 Division Heart & Lungs, Department of Cardiology, University Medical Center Utrecht, Utrecht University, Utrecht, the Netherlands; 3 Department of Clinical Sciences, Faculty of Veterinary Medicine, Utrecht University, Utrecht, the Netherlands; 4 Department of Nephrology and Hypertension, University Medical Center Utrecht, Utrecht University, Utrecht, the Netherlands; 5 Department of Physiology, Amsterdam UMC, Location VUmc, Amsterdam, the Netherlands; 6 Cardiovascular Research Institute Maastricht (CARIM), Maastricht, the Netherlands

**Keywords:** Cell culture, Cell-based Assays, Tissue Engineering

## Abstract

Here, we present a protocol for building a vascularized heart-on-a-chip by co-encapsulating human induced pluripotent stem cell (hiPSC)-derived cardiomyocytes and endothelial cells in vascular endothelial growth factor (VEGF)- and aprotinin-supplemented fibrin. We describe steps for cell preparation, fibrin formulation, chip loading, optional droplet-based pilot or high-throughput setups, and imaging. We then detail procedures for immunofluorescence staining and routine quantitative analysis of vascular and contractile features during culture for up to three weeks.

## Highlights

• Steps to co-encapsulate endothelial cells and cardiomyocytes in a fibrin hydrogel
• Details to load hydrogel-cell mixture into a commercial microfluidic chip
• Instructions for performing immunofluorescence staining within the intact microfluidic chip
• Steps to quantify vascular networks and cardiomyocyte contraction with open-source tools

## Abstract (editor highlights)

Publisher’s note: Undertaking any experimental protocol requires adherence to local institutional guidelines for laboratory safety and ethics.

## Graphical abstract

**Figure:**
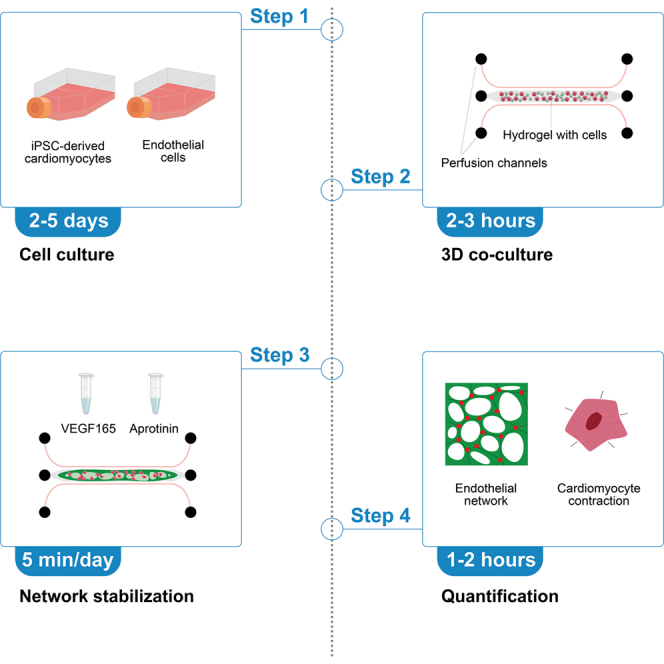

## Before you begin

The following steps describe the preparation of endothelial cells and human iPSC-derived cardiomyocytes, as well as the essential supplements required to establish the co-culture in a microfluidic device. The workflow is compatible with alternative chip geometries that contain a central gel compartment. We have successfully used seeding cell densities ranging from 3–100 × 10^6^ cells per mL for each cell type. However, in our experience, nutrient supply and gas exchange become limiting in sealed devices at the upper end of this range, resulting in impaired long-term culture. Therefore, for most applications, we recommend using 6–15 × 10^6^ cells per mL for each cell type.

### Preparations

1. Prepare coatings. Use gelatin for human umbilical vein endothelial cells (HUVECs) and Matrigel for hiPSC-derived cardiomyocytes.
2. Prepare endothelial cells at passage ≤7. In this protocol, we used HUVECs labelled with green fluorescent protein (GFP).
3. Thaw hiPSC-derived cardiomyocytes and allow sufficient recovery before co-culture.
4. Prepare stock solutions of thrombin, fibrinogen, and VEGF165.
5. Clean and prepare the microfluidic device and tubing.

***Note:*** This protocol is optimized for loading fibrin-based co-cultures into microfluidic chips. For users who wish to perform initial optimization or generate large numbers of replicates, the hydrogel-cell suspension can alternatively be cast as simple fibrin droplets. This droplet alternative follows the same workflow up to Step 9 and is described under.

***Optional:*** Preparation of fibrin co-culture droplets in the Gel loading and polymerization section.

***Note:*** Channel dimensions of the microfluidic chip are available in the Fluidic 983 technical datasheet (microfluidic ChipShop).

***Note:*** Additional supporting cell types can be incorporated if desired. For example, bone marrow-derived stromal cells (BMSCs) can replace hiPSC-derived cardiomyocytes and form stable co-cultures with endothelial cells.^1^

***Note:*** This protocol was validated using two independent hiPSC-derived cardiomyocyte lines. HUVECs were obtained from different donors (pooled cell line) and used before passage 7.

**CRITICAL:** Use sterile techniques throughout device preparation, gel mixing, and loading to prevent contamination.

### Innovation

This protocol presents an accessible vascularized heart-on-chip model for cardiac disease modeling and drug response studies, integrating cost-efficient and openly available components, controlled unidirectional perfusion, and culture conditions that support long-term vascular network stability.

Cardiac co-culture models and organ-on-chip platforms have advanced considerably, yet many still require specialized equipment, complex handling procedures, or custom-built microfluidic devices, limiting accessibility and experimental throughput.^2^^,^^3^^,^^4^ Many also rely on polydimethylsiloxane (PDMS)-based devices that absorb hydrophobic molecules and small compounds, complicating reproducibility and drug testing.^2^

Our approach uses a commercially available PDMS-free microfluidic device combined with a fibrin-based endothelial-cardiomyocyte co-culture system, reducing absorption-related concerns, avoiding the need for custom microfabrication, and supporting robust vascular network formation. All components and reagents are openly available without dependence on a closed proprietary workflow, facilitating adoption across laboratories.

The microfluidic chip enables controlled unidirectional perfusion for sustained nutrient delivery and waste removal within a defined three-dimensional architecture. The central gel compartment supports spatially organized endothelial-cardiomyocyte co-cultures with sustained cell-cell interactions and enables formation of lumenized microvascular networks with diameters comparable to native human microvasculature, typically <20 μm.^5^ The device is optically clear, allowing direct three-dimensional imaging of vessels and cardiomyocyte structure without additional embedding or tissue clearing.

By combining VEGF signalling with protease inhibition (aprotinin) and optimized culture conditions, endothelial self-assembly into vascular networks that remain stable for at least three weeks was achieved in static droplet format, exceeding the typical one to two week culture durations reported for conventional *in vitro* endothelial network assays.^6^

### Institutional permissions

Perform all procedures in accordance with local institutional biosafety and ethical regulations for human cell lines. In our case, the work was conducted in an ML1 laboratory in accordance with approved institutional guidelines. Induced (reprogrammed) cell lines were downscaled to ML1 in accordance with directives issued by national regulatory safety authorities. Authorization for the use of human cell lines should be obtained through the appropriate institutional committee, and material transfer agreements (MTAs) must be in place. Readers must obtain equivalent approvals from their own institutions before starting the protocol.

### Preparation 1: Cleaning of the device and tubing

**Timing: ∼20 min**

This section describes the cleaning and sterilization of the microfluidic chip and perfusion components prior to use. Thorough removal of manufacturing residues and potential contaminants is necessary to prevent interference with cell culture.

6. Clean the device
  a. Flush each channel of the Fluidic 983 chip with 70% ethanol, then incubate for 10 min.
  b. Rinse each channel twice with sterile PBS.
  c. Remove all residual liquid from the channels by using an aspiration system.

***Note:*** Although the chips are manufactured in cleanroom conditions, they should never be considered sterile. Therefore, ethanol cleaning is recommended.

***Optional:*** Expose the chip to germicidal UV for 20 min in a laminar flow hood.

7. Clean the perfusion tools
  a. Flush tubing with 70% ethanol and ensure a contact time of 10 minutes.
  b. Immerse all other perfusion components in 70% ethanol for 10 min.
  c. Thoroughly flush the tubing with sterile PBS to remove any residual traces of ethanol, and rinse all components twice with sterile PBS.
  d. Remove PBS from all hollow components to prevent air pockets from forming during subsequent perfusion steps. Apply gentle positive pressure with a filtered gas line or carefully aspirate using a vacuum aspiration system.

### Preparation 2: Cell recovery and expansion

**Timing: Typically 2**–**5 days**

This section covers the thawing and culture of HUVECs and hiPSC-derived cardiomyocytes in preparation for co-culture. Both cell types require sufficient recovery time after thawing to ensure adequate viability and cell numbers before proceeding to hydrogel loading.

**Figure 1:**
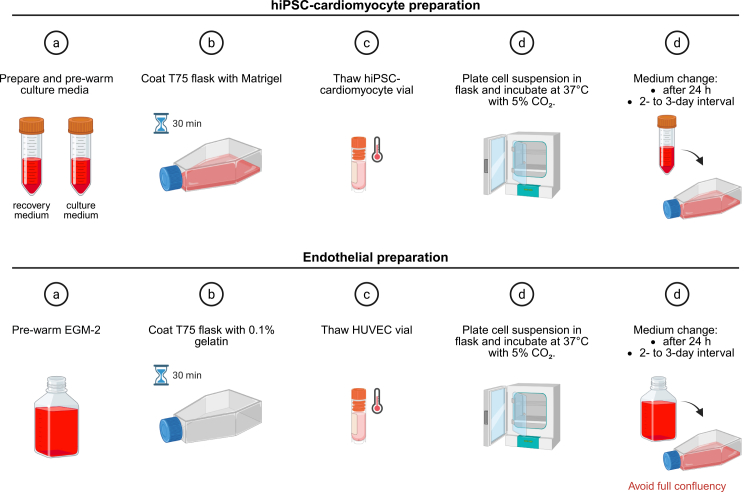
Schematic overview of Preparation 2: Cell recovery and expansion

A schematic overview is shown in Figure 1.

8. Thaw and optionally expand hiPSC-derived cardiomyocytes according to a previously published protocol.^7^
  a. Prepare cardiomyocyte recovery and culture media and pre-warm to 37°C.
  b. Coat a T75 flask with Matrigel at 0.1 mg per mL for at least 30 min at 37°C.
  c. Quickly thaw the vial in a 37°C water bath, dilute in warm RPMI, centrifuge at 200 × *g* for 3 min, and resuspend in recovery medium.
  d. Remove the Matrigel solution from the flask, add the cell suspension, and incubate at 37°C and 5% CO_2_.
  e. Replace with cardiomyocyte culture medium after 24 h, and continue 2- to 3-days interval medium changes until initiation of the co-culture.
9. Thaw HUVECs.
  a. Pre-warm EGM-2.
  b. Coat a T75 flask with 3 mL 0.1% gelatin for at least 30 min at 37°C.
  c. Quickly thaw the vial in a 37°C water bath until only a small frozen unit can be seen and dilute the DMSO into prewarmed 15 mL EGM-2.
  d. Remove gelatin from the flask, add cell suspension, and incubate at 37°C and 5% CO_2_.
  e. Replace the medium after 24 h, and continue 2- to 3-days medium changes until co-culture has set. Keep the passage number to a maximum of 7.

**Pause point:** Both cell types can be maintained in culture medium with regular medium changes for several days. Proceed to dissociation before HUVECs reach full confluency.

***Note:*** Wear personal protective equipment when handling liquid nitrogen and cryovials.

***Note:*** Routinely inspect cultures under a tabletop microscope for morphology, confluency, excess cell death, and contamination.

***Note:*** hiPSC-derived cardiomyocytes typically show limited proliferation in standard culture medium. If active cardiomyocyte expansion is desired, proliferation can be promoted by supplementing the culture medium with freshly prepared 2 μM CHIR99021.^7^

**CRITICAL:** Avoid full confluency in endothelial cultures, as it has been shown to alter VEGF receptor signaling.^8^

## Key resources table

**Table:** 

REAGENT or RESOURCE	SOURCE	IDENTIFIER
**Antibodies**		

alpha-Actinin (Sarcomeric) monoclonal mouse (1:200)	Sigma-Aldrich	A7811 RRID:AB_476766
Donkey anti-Mouse IgG (H+L) Highly Cross-Adsorbed Secondary Antibody, Alexa Fluor™ 568 (1:400)	Thermo Fisher Scientific	A10037 RRID:AB_11180865

**Chemicals, peptides, and recombinant proteins**		

Fibrinogen from human plasma	Sigma-Aldrich	341576
Thrombin from bovine plasma	Sigma-Aldrich	T4648
Aprotinin	Sigma-Aldrich	A6279
VEGF165	Peprotech	AF-100-20
RPMI 1640	Thermo Fisher	11875093
B27+ supplement	Gibco	17504–044
Penicillin/Streptomycin	Sigma-Aldrich	P4333
KnockOut serum replacement	Gibco	10828–028
Revitacell	Gibco	A2644501
CHIR-99021	Selleck Chemicals	S2924
EGM-2 medium	Lonza	CC-3162
Matrigel Growth Factor Reduced (GFR) Basement Membrane Matrix, LDEV-free	Corning	356230
TrypLE Express Enzyme (1X), no phenol red	Gibco	12604039
TrypLE Select Enzyme (10X), no phenol red	Gibco	A1217701
Gelatin powder	Sigma-Aldrich	G1890
DPBS, no calcium, no magnesium	Gibco	14190–144
Bovine Serum Albumin (BSA)	Sigma-Aldrich	A7888
DAPI	Thermo Scientific	62248
PFA 4%	Thermo Scientific	J61899.AK
Triton X-100	Sigma-Aldrich	X100
CellTracker Deep Red	Invitrogen	C34565

**Experimental models**: Cell lines		

Human hiPSC-derived cardiomyocytes, healthy male donor	Obtained from collaborator (Prof J. van der Velden, Amsterdam University Medical Center).	Not applicable (collaborator-derived)
HUVECs GFP-labelled, human, mixed donors	Obtained from collaborator (Dr. C. van Dijk, Regenerative Medicine Center Utrecht). Commercial alternative: Lonza, ATCC, PromoCell	Not applicable (collaborator-derived)

**Software and algorithms**		

MuscleMotion ImageJ plugin	Sala et al.^9^	https://github.com/l-sala/MUSCLEMOTION
Angiotool 2.0		https://github.com/jbendtsen/AngioTool-Batch
Fiji/ImageJ	Schneider et al.^10^	https://imagej.net/software/fiji/downloads
FFmpeg plugin		https://imagej.net/plugins/ffmpeg-video-import-export

**Other**		

Fluidic 983 Channel Interaction Chip. Zeonor, no surface treatment.	Microfluidic chipshop	10001349
Male Mini Luer plugs – Low volume displacement	Microfluidic chipshop	10000280
Silicone tube, ID: 0.76 mm, OD: 1.65 mm	Microfluidic chipshop	10000031
Micro tubes, PTFE, ID: 0.5 mm, OD:1.0 mm	Microfluidic chipshop	10000032
Male Mini Luer fluid connector	Microfluidic chipshop	10000116
Optional: 500 μl sampling vessels	Microfluidic chipshop	10000761
Anaesthesia pump	Graseby	3500
Cell counter TC20	Biorad	

## Materials and equipment

### Stock solutions

• **Fibrinogen solution**

Reconstitute 100 mg fibrinogen in 20 mL calcium- and magnesium-free PBS at 37°C to obtain a 5 mg/ml solution. Filter the solution through a low-protein-binding 0.22 μm filter. Aliquot, snap freeze on dry ice, and store aliquots at −80°C.

• **Thrombin solution**

Dissolve thrombin at 100 U/ml in PBS with 1% BSA. Sterilize the solution with a low-protein-binding 0.22 μm filter. Aliquot the sterile solution in plastic tubes and store at −20 °C for long-term storage.

• **VEGF165 solution**

Reconstitute VEGF165 to 100 μg/mL in PBS with 1% BSA, aliquot, and store at −80°C.

### Coating solutions

• **Gelatin solution 0.1% for surface coating**

Dissolve gelatin at 0.1% (w/v) in Milli-Q water and bring to the desired final volume. Sterilize the solution by autoclaving for 15 min at 121°C. Allow to cool and store at 20-25°C. Once opened, store the solution at 4°C.

• **Matrigel stock solution (1.2 mg/mL in RPMI 1640)**

Thaw Matrigel 12-16 h at 4°C. Keep all materials on ice by using small ice containers. In a sterile hood, dilute the thawed Matrigel to 1.2 mg/mL in cold RPMI 1640. Aliquot 1 mL into prechilled 15 mL tubes and store at −20°C. Avoid repeated freeze-thaw cycles.

• **Matrigel working solution (0.1 mg/mL)**

Thaw one 1 mL stock aliquot on ice and dilute with 11 mL cold RPMI 1640. Keep the working solution on ice and use it within 3 days.

### Cell culture media

• **Cardiomyocyte culture medium**

Prepare RPMI 1640 supplemented with B27 Plus (1:50) and penicillin-streptomycin (1:100). Store at 4°C for up to one week. If cardiomyocyte expansion is desired, add 2 μM CHIR99021 fresh to the medium immediately before use.^7^

**Table:** 

Reagent	Final concentration	Amount
RPMI 1640	N/A	To 50 mL
B27 Plus	1:50	1 mL
Penicillin-streptomycin	1:100	0.5 mL
Optional: CHIR99021	2 μM	2.5 μL of 40 mM stock
**Total**	**N/A**	**50 mL**

• Cardiomyocyte replating medium

Prepare RPMI 1640 supplemented with KOSR (1:10), B27 Plus (1:50), RevitaCell (1:100), and penicillin-streptomycin (1:100). Prepare fresh on the day of use.

**Table:** 

Reagent	Final concentration	Amount
RPMI 1640	N/A	To 50 mL
KOSR	1:10	5 mL
B27 Plus	1:50	1 mL
RevitaCell	1:100	0.5 mL
Penicillin-streptomycin	1:100	0.5 mL
**Total**	**N/A**	**50 mL**

• **Endothelial cell culture medium**

Prepare EGM-2 by supplementing EBM-2 basal medium with the provided EGM-2 SingleQuots. Optionally supplement with extra antibiotics by adding penicillin-streptomycin (1:100). Store at 4°C for up to one month.

• **Co-culture recovery medium**

Mix EGM-2 and cardiomyocyte replating medium in a 1:1 ratio (v/v). Prepare freshly on the day of use.

• **Co-culture medium**

Mix EGM-2 and cardiomyocyte culture medium in a 1:1 ratio (v/v) and supplement with VEGF (25 ng/mL) and aprotinin (1:300; effective end concentration 0.01–0.07 TIU). Prepare freshly prior to use.

**Table:** 

Reagent	Final concentration	Amount
EGM-2	1:2	25 mL
Cardiomyocyte culture medium	1:2	25 mL
VEGF	25 ng/mL	12.5 μL of 100 μg/mL stock
Aprotinin	1:300; effective end concentration 0.01–0.07 TIU	166.7 μL
**Total**	**N/A**	**approximately 50 mL**

***Note:*** Both VEGF signalling and fibrinolysis inhibition are dose-dependent and jointly determine endothelial network formation and stability in fibrin matrices. Fibrin vasculogenesis models commonly use VEGF supplementation in the 25–50 ng/ml range to promote robust endothelial self-assembly and network connectivity.^1^^,^^6^^,^^11^ Accordingly, we selected 25 ng/ml VEGF as a moderate concentration that supports network formation without excessively accelerating sprouting and matrix remodelling. To limit fibrin degradation while avoiding overt suppression of vascular morphogenesis, we added aprotinin at 0.01 to 0.07 trypsin inhibitory units (TIU). This falls within the low-to-intermediate range of protease inhibition reported to modulate fibrin stability and network morphology.^6^^,^^12^

***Note:*** The manufacturer specifies an activity range of 3–21 TIU/mL for the aprotinin stock solution, resulting in an effective end concentration of 0.01–0.07 TIU/mL.

### Staining solution

• **Permeabilization solution**

Prepare the permeabilization solution by weighing 5 g of BSA and adding it to 100 mL of PBS. Allow the BSA to dissolve at 4°C without mixing or mix with very gentle low-speed agitation to prevent foam formation. Once the BSA is fully dissolved, add 300 μL Triton X-100 and mix until homogeneous. Store at 4°C for up to one week.

**Table:** 

Reagent	Final concentration	Amount
BSA	5% (w/v)	5 g
PBS	N/A	100 mL
Triton X-100	0.3% (v/v)	300 μL
**Total**	**N/A**	**approximately 100 mL**

## Step-by-step method details

### Dissociate and count HUVECs and hiPSC-derived cardiomyocytes

**Timing: 30**–**45 min**

This section describes the enzymatic dissociation of HUVECs and hiPSC-derived cardiomyocytes from their culture flasks and the preparation of single-cell suspensions for co-culture. Accurate cell counting at this stage is essential for calculating the volumes needed to achieve the target seeding densities in the hydrogel.

1. Aspirate the culture medium and wash each flask once with calcium- and magnesium-free PBS.
2. For HUVECs:
  a. Add an appropriate volume of TrypLE Express (e.g., 2.5 mL for a T75 flask) to fully cover the monolayer.
  b. Incubate at 20–37°C and monitor under an inverted microscope until the cells round up.
  c. Gently tap the flask to release the cells, then stop enzymatic activity by adding 8 ml of warm EGM-2 medium.
  d. Collect the cell suspension into a 15 mL tube and centrifuge at 200 × *g* for 5 min.
  e. Discard the supernatant and resuspend the pellet gently in 0.5–1 mL EGM-2.
3. For hiPSC-derived cardiomyocytes:
  a. Add an appropriate volume of TrypLE Select (e.g., 2.5 mL for a T75 flask) to cover the cells. Distribute evenly by tilting the flask.
  b. Incubate at 37°C and regularly check until detachment is observed.
  c. Collect the cells, immediately dilute in 8 mL of warm RPMI medium, and centrifuge at 200 × *g* for 3 min.
  d. Discard the supernatant and resuspend the pellet gently in 0.5–1 mL replating medium.
4. Count both suspensions using trypan blue exclusion and record viability.

**CRITICAL:** Avoid over-digestion and minimize mechanical stress to preserve cell viability.

***Note:*** Low cell viability can compromise subsequent co-culture performance. Use only cell suspensions with a viability above 60% to avoid introducing excessive dead cells into hydrogel.

***Optional:*** Instead of HUVECs-GFP, live-cell dyes can be used to track endothelial cells and quantify network morphology. We have successfully used CellTracker in different colors on both HUVECs and hiPSC-derived cardiomyocytes. This approach is particularly useful for hiPSC-derived cardiomyocytes, as they proliferate minimally and maintain dye intensity over time.

Live dyes can be cytotoxic at higher concentrations. We recommend optimizing the concentration for each cell type before use. Proliferation or viability stainings can be used to evaluate toxic effects. In our hands, optimal labeling of 1 million endothelial cells and hiPSC-derived cardiomyocytes was achieved by using 2.5 μM CellTracker in 1 ml of protein-free buffer, incubated for 15 min at 20–25°C.

### Prepare fibrin co-culture suspension and load the gels

**Timing: 30**–**45 min**

This section covers the preparation of the fibrin-based co-culture suspension by combining the two cell types at defined densities and adding thrombin and VEGF. The resulting suspension serves as the input for gel loading into the microfluidic chip or, alternatively, for casting as fibrin droplets.

5. Calculate the required number of HUVECs and hiPSC-CMs for a 1:1 mixture, targeting a final concentration of 6.0–15.0 × 10^6^ live cells/mL. Use Table S1.
6. Mix the calculated amounts of HUVECs and hiPSC-derived cardiomyocytes in a 1.5 ml tube and centrifuge at 200 × *g* for 3 min.
7. Resuspend the combined pellet in EGM-2 to the required volume.
8. Add thrombin to a final concentration of 2 U/mL and VEGF to a final concentration of 0.1 μg/mL.

***Note:*** A tabletop mini-centrifuge can be used to pellet cells in 1.5 mL microcentrifuge tubes. These tubes are preferred over 15 mL conical tubes because the small suspension volume facilitates easier handling and more accurate supernatant removal.

### Gel loading and polymerization

**Timing: 15 min**

This section describes the mixing of the cell suspension with fibrinogen and the loading of the hydrogel into the central channel of the microfluidic chip. Because fibrin polymerization begins immediately upon contact with thrombin, this step requires rapid handling to ensure even gel distribution before the mixture solidifies.

**Figure 2:**
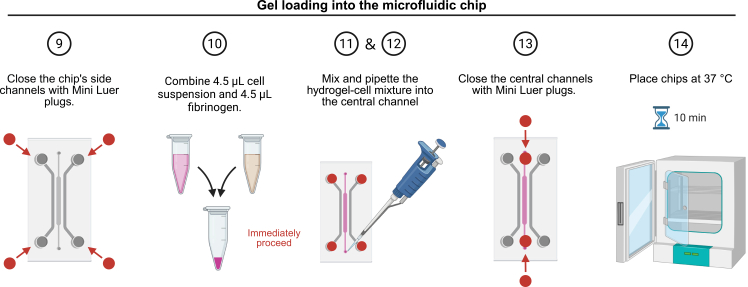
Schematic overview of step 9–14: Gel loading and polymerization

A schematic overview is shown in Figure 2.

9. Close the chip’s side channels with low-displacement Mini Luer plugs.
10. In a sterile 1.5 mL microcentrifuge tube, combine 4.5 μL cell suspension and 4.5 μL fibrinogen. Proceed rapidly, as thrombin in the cell suspension initiates fibrin polymerization upon mixing.
11. Keep the pipette setting on 4.5 μL and mix gently by pipetting up and down 3 times while keeping the pipette tip submerged in the liquid to avoid air bubbles.
12. Immediately aspirate 8.5 μL and pipette the hydrogel-cell mixture into the central channel.
13. Close the central channels with low-displacement Mini Luer plugs.
14. Place chips at 37 °C for 10 minutes to allow polymerization.

**CRITICAL:** Load a freshly prepared gel mixture for each lane, as fibrin polymerization begins approximately 1 min after mixing with thrombin. Delays may cause partial gelation and clogging of the pipette tip.

***Optional:*** To minimize handling time, prepare two pipettes in advance: one set to 4.5 μL for mixing and one set to 8.5 μL for loading, so that the gel can be transferred into the chip immediately after preparation.

***Optional:*** Instead of loading the hydrogel-cell mixture into the microfluidic chip, the same suspension can be cast as simple fibrin droplets for optimization experiments or to obtain large numbers of replicates. After preparing the hydrogel mixture as described above, dispense a small volume (for example, 10 μL) onto a standard tissue-culture-treated plastic surface or a glass-bottom dish. Avoid highly hydrophilic or coated surfaces, such as gelatin-coated wells, as they can cause the droplet to lose its shape. Allow the droplet to polymerize for 10 min at 37°C. Once the gel has set, gently add co-culture medium along the edge of the well to prevent the droplet from detaching. Maintain droplets in co-culture medium supplemented with VEGF and aprotinin as described for chip culture.

### Culture conditions

**Timing: 30 min initial, 10 min per day for media changes**

This section describes the initiation and maintenance of the co-culture after gel loading. It covers medium changes, perfusion setup, and incubation conditions required to support endothelial network formation and cardiomyocyte viability over the culture period.

15. Connect the tubing or reservoirs to the chip and fill the lateral channels with co-culture recovery medium.
16. Maintain at 37°C, 5% CO_2_.
17. On day 1, replace the medium with co-culture medium.

**CRITICAL:** Avoid bubble formation, as air bubbles can block medium flow and disrupt perfusion. Degassing the medium before use is recommended to minimize bubble formation. As a simple manual degassing method, place the medium in a syringe, seal the outlet, and gently withdraw the plunger to create negative pressure.

**CRITICAL:** When using static reservoirs, replace the medium at least twice daily to ensure a sufficient nutrient supply.

When using a perfusion pump, initiate perfusion immediately at a flow rate of 2 μL/min. This low flow rate provides gentle interstitial perfusion to support nutrient exchange and vessel maintenance while minimizing the risk of gel displacement and the need for syringe changes that may introduce air bubbles. Perfuse medium through the lateral channels in parallel with the central hydrogel chamber. Do not apply pressure across the hydrogel to allow the medium to flow along the central chamber rather than through the gel. Once perfusable vessels have formed, typically after three days, controlled flow through the central channel becomes feasible. See Methods video S1 for a representative video of a co-culture after 3 days.

Methods Video S1. Brightfield video of a co-culture of endothelial cells and hiPSC-derived cardiomyocytes cardiomyocytes at day 3 within the microfluidic chip Both cell types were seeded at 9.0 × 10^6^ cells/mL each. Scalebar, 250 μm. Related to step 17.

### Immunofluorescence staining

**Timing: 3**–**4 days (including antibody incubations)**

This section describes the fixation, permeabilization, and antibody staining of co-cultures within the intact microfluidic chip. The procedure enables three-dimensional visualization of endothelial networks and cardiomyocyte sarcomere organization without the need for embedding or sectioning.

18. Fix the samples.
  a. Remove all tubing.
  b. Aspirate medium via the side-channel inlets.
  c. Rinse channels twice with calcium- and magnesium-free PBS. Allow fluids to pass slowly through the chip to minimize shear forces and the chance to flush the matrix from the central channel.
  d. Introduce 4% paraformaldehyde (PFA) and incubate for 15 min at 20–25°C.
  e. Flush three times 800 μL PBS through the chip using reservoirs to ensure complete removal of fixative.
  f. Store chips at 4°C with reservoirs attached or closed with Mini Luer plugs to prevent drying until staining.

**Pause point:** Fixed chips can be stored in PBS at 4°C for up to one week before proceeding to staining. For storage beyond a few days, adding 0.02% sodium azide to the PBS is recommended to prevent microbial growth.

19. Permeabilize and block: Introduce permeabilization solution (PBS containing 5% BSA and 0.3% Triton X-100) into the channels and incubate for 60 min at 20–25°C.
20. Incubate with primary antibody.
  a. Prepare anti-alpha actinin-2 (ACTN2) diluted 1:200 in five times diluted permeabilization solution (1% BSA and 0.06% Triton X-100).
  b. Spin the antibody solution at full speed in a tabletop centrifuge for 10 min, then pipette from the top to avoid collecting aggregates of antibody or stain.
  c. Use 200 μL antibody solution per chip.
  d. Incubate chips for 24 h at 4°C in a humidified container, placed on a rocker. Orient the chip such that opposing pillar levels change during the cycle of the rocker, and rock slowly to minimize shear forces.
21. Wash chips three times with 400 μL PBS for 30 min each at 4°C.
22. Incubate with secondary antibody.
  a. Dilute donkey anti-mouse Alexa Fluor 568 1:400 in five times diluted permeabilization solution.
  b. Spin the antibody solution at full speed in a tabletop centrifuge for 10 min, then pipette from the top to avoid collecting aggregates of antibody or stain.
  c. Introduce 200 μL solution per chip and incubate for 48 h or over the weekend at 4°C in the dark, in a humidified container placed on a rocker.
23. Wash chips twice with 400 μL PBS for 30 min each at 4°C.
24. Counterstain nuclei.
  a. Dilute DAPI 1:1000 in 200 μL PBS.
  b. Incubate chips for 15 min at 20–25°C.
  c. Wash once with 400 μL PBS for 30 min at 4°C.

**CRITICAL:** Ensure humidity inside the container to prevent evaporation of the antibody solution inside the chip.

## Expected outcomes

**Figure 3:**
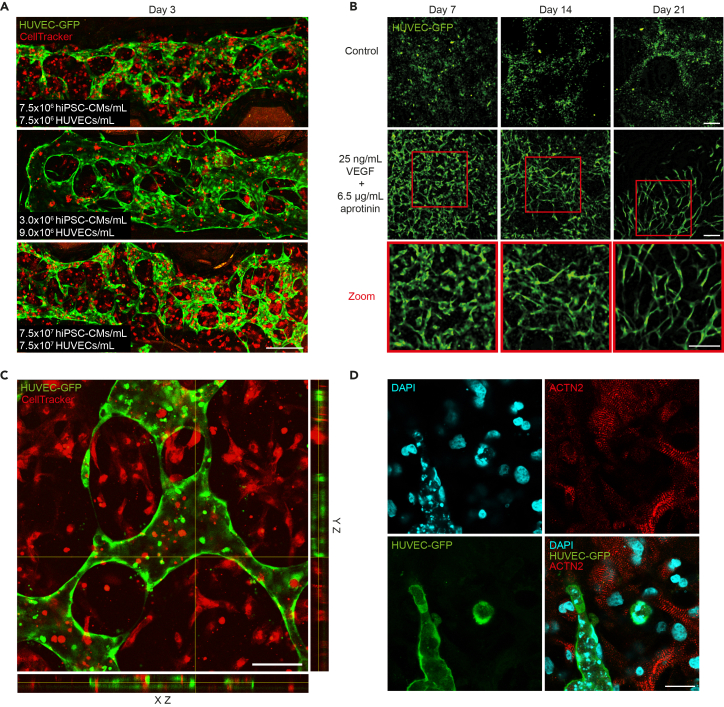
Endothelial-cardiomyocyte co-culture in fibrin hydrogels (A) Representative networks at day 3 across different starting densities of hiPSC-derived cardiomyocytes (hiPSC-CMs) and human umbilical vein endothelial cells (HUVECs) within a chip. hiPSC-derived cardiomyocytes and endothelial cells were seeded at 7.5 × 10^6^ cells/mL each (top), 3.0 × 10^6^ and 9.0 × 10^6^ cells/mL (middle), and 1.5 × 10^7^ cells/mL each (bottom). HUVECs are shown in green (GFP) and cardiomyocytes in red (CellTracker Deep Red). Scale bar, 250 μm. (B) Effect of VEGF and aprotinin on network stability in static fibrin droplets. HUVEC-GFP fluorescence at days 7, 14, and 21 in control conditions (top row) and in cultures supplemented with 25 ng/mL VEGF and aprotinin (middle row). Red boxes indicate areas shown at higher magnification (bottom row). Seeding concentrations were 9 × 10^6^ CMs/mL and 6 × 10^6^ ECs/mL. Scale bar, 100 μm. (C) Lumen formation in endothelial-cardiomyocyte co-culture within a chip. Orthogonal views through the confocal z-stack are shown along the x-z axis (bottom) and y-z axis (right). The yellow lines indicate the position of the single x-y image plane shown in the central panel. HUVEC-GFP is shown in green and hiPSC-CMs in red (CellTracker Deep Red). The hollow profiles visible in the orthogonal views indicate lumen formation. Scale bar, 100 μm. (D) Immunofluorescence staining of co-cultures within a chip at day 7. Nuclei (DAPI, cyan), endothelial cells (HUVEC-GFP, green), and cardiomyocyte marker ACTN2 (red). Scale bar, 25 μm. See Methods Video S2 for z-stack montage showing the spatial distribution of HUVECs and hiPSC-CMs across the gel depth.

**Figure 4:**
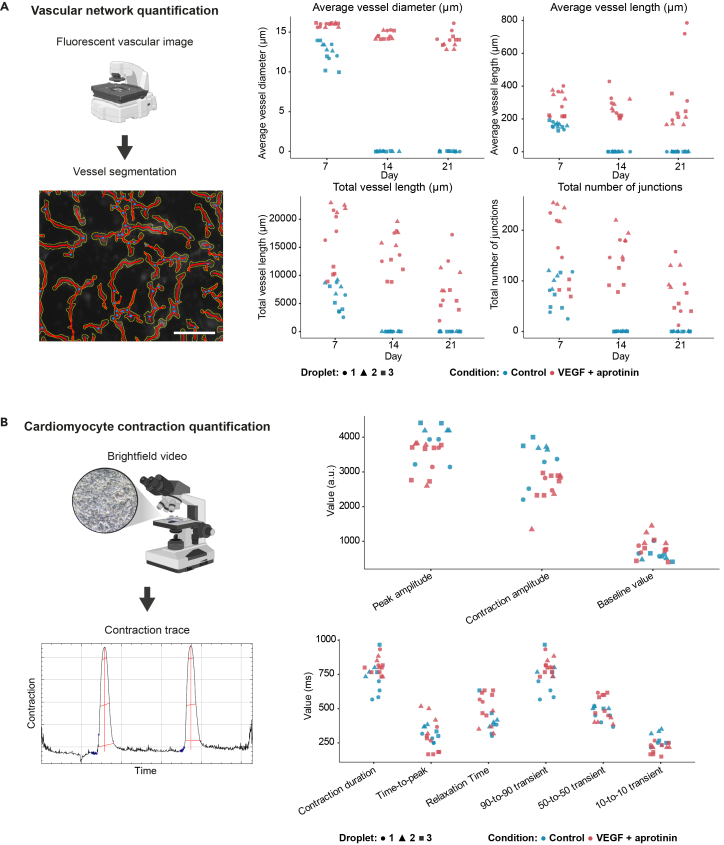
Quantitative analysis of vascular networks and cardiomyocyte contraction (A) Quantification of endothelial networks in fibrin droplets. Fluorescent vascular images were segmented with the ImageJ/Fiji plugin AngioTool 2.0 to extract vessel geometry. Representative example showing the raw image (grayscale) with segmentation overlay, including the detected vessel skeleton (red) and vessel outline (yellow), which together illustrate the basis for vessel diameter and network quantification. Scalebar, 100 μm. Plots show average vessel diameter, average vessel length, total vessel length, and total number of junctions at days 7, 14, and 21 in control conditions and in cultures supplemented with VEGF and aprotinin. Data points represent quantification from 12 image areas (4 areas per droplet, 3 droplets) per condition and time point, from a single experiment. Different symbols indicate individual droplets. Data are presented descriptively to illustrate expected outcomes; no statistical tests were performed. (B) Quantification of cardiomyocyte contraction at day 5 in fibrin droplets. Brightfield videos were analyzed to extract contraction traces with the ImageJ/Fiji plugin MUSCLEMOTION. An example trace shows contraction peaks and derived parameters. Plots show peak amplitude, contraction amplitude, beating rate, contraction duration, time to peak, relaxation time, and 90% transient durations under control conditions and under VEGF plus aprotinin. Each point represents a detected contraction peak. Contraction measurements from droplets treated with VEGF and aprotinin overlap with the measurements in the control conditions.

This protocol generates stable endothelial-cardiomyocyte co-cultures in which endothelial cells consistently form interconnected and perfusable vascular networks across a broad range of starting cell densities and mixing ratios. Figures 3 and 4 present data following this protocol. Networks typically develop throughout the fibrin hydrogel and remain continuous even when initial cell densities range from 3.0 × 10^6^ to 15.0 × 10^6^ cells per milliliter (Figure 3A). We recommend starting with 7.5 × 10^6^ cells per milliliter from each cell type. In higher-density preparations, network formation remains feasible, although, in our experience, nutrient diffusion can restrict long-term stability in closed systems.

Methods Video S2. Z-stack animation of immunofluorescence staining of co-cultures within a chip at day 7 Nuclei (DAPI, cyan), endothelial cells (HUVEC-GFP, green), and cardiomyocyte markers ACTN2 (red) and TNNI3 (white). Z-stack comprises 12 optical sections with a 0.43 μm step size (5.1 μm total depth). Related to step 18–24.

In static co-culture droplets, vascular structures persist for at least three weeks when supplemented with VEGF and aprotinin. In the absence of these factors, endothelial networks progressively regress (Figure 3B). Z-stack imaging of cultures in the microfluidic chip confirms the presence of hollow luminal structures within the formed vessels (Figure 3C), demonstrating maturation beyond simple endothelial cords and addressing a common limitation observed in endothelial co-culture systems.^13^ In the perfused chip format, co-cultures were maintained for several days; extended perfusion durations are limited by bubble accumulation.

hiPSC-derived cardiomyocytes remain structurally intact within the co-culture and maintain organized sarcomere architecture. Spontaneous beating is typically observed during the first week of culture, after which the activity often decreases, a pattern commonly noted as hiPSC-derived cardiomyocytes progress toward a more mature phenotype.^14^ Structural staining with alpha-actinin (ACTN2) demonstrates preserved sarcomere organization and allows assessment of cellular alignment and interactions with adjacent vessels (Figure 3D).

A key advantage of this system is that the microfluidic chip is thin and optically clear, allowing direct 3D imaging of the tissue without embedding, sectioning, or clearing. This facilitates high-resolution visualization of endothelial and cardiomyocyte features in their native spatial arrangement. Additional antibody stainings can also be performed within the intact chip, enabling three-dimensional assessment of molecular targets, cellular interactions, and spatial architecture throughout the engineered tissue.

## Quantification and statistical analysis

Quantify vascular networks by acquiring fluorescent images at 10x or 20x magnification using identical excitation and exposure settings for all samples. For live cultures, maintain a low fluorescent excitation intensity (≤20% of maximum) and minimize exposure time to limit phototoxicity. Capture multiple fields per chip or droplet to account for local variation. Analyze images with AngioTool 2.0, an adaptation of the original AngioTool.^15^ AngioTool 2.0 runs as a standalone Java.jar application compatible with macOS, Windows, and Linux. Consult the AngioTool 2.0 manual to guide parameter selection. The AngioTool 2.0 settings used to generate the data presented in Figure 4A are provided in Table S2. First, optimize detection settings on a representative subset of images, then apply the finalized parameters to the full dataset in batch mode. AngioTool outputs an Excel file containing explant area, vessel area, and percentage area, total and average vessel length, total and density of junctions and endpoints, average vessel diameter, and lacunarity-based measures of network complexity. Exclude images that are oversaturated or out of focus.

Quantify cardiomyocyte contraction by recording videos at 10x or 20x magnification in brightfield with a minimum frame rate of 60 frames per second. Capture several consecutive beats for stable measurements. When dedicated high-frame-rate acquisition is not available on the microscope, use a smartphone with a microscope phone adapter or an action camera with an adapter as an alternative. Convert the recordings to an ImageJ-compatible MJPEG-encoded AVI using the FFmpeg Video plugin. Analyze the videos using the MUSCLEMOTION plugin^9^ in Image,^10^ following the developer’s manual for guidance. MUSCLEMOTION provides baseline and peak intensity values, contraction amplitude, time to peak, relaxation time, transient durations, and the peak-to-peak interval as a measure of beating frequency. Exclude recordings affected by motion artefacts or moving debris in the field of view. Batch processing using ImageJ macros is compatible with this workflow. The MUSCLEMOTION settings used to analyze the videos underlying Figure 4B are provided in Table S3.

## Limitations

This protocol uses HUVECs as the endothelial component, which introduces two key limitations. First, HUVECs are not cardiac-specific and are derived from veins instead of capillaries, the desired blood vessel type in this project, and therefore lack organ and vascular tree hierarchy-specific endothelial characteristics. Endothelial cells retain organ-dependent transcriptional and functional programs, even after several passages in culture.^16^ Second, HUVECs are not patient-specific, limiting applicability in personalized disease modeling. While patient-derived iPSC endothelial cells could address this limitation, robust differentiation and validation strategies that reliably capture cardiac endothelial identity are not yet standardized for routine use in this workflow.

Our model includes only endothelial cells and hiPSC-derived cardiomyocytes. Adding additional cell types, such as pericytes and fibroblasts, would improve vascular stability and functionality.^17^^,^^18^ Although the chip design technically supports adding other cell types, increased cellular complexity reduces reproducibility and lowers throughput.

Functional perfusability of the self-assembled vascular network was not validated (e.g., by tracer perfusion). Lumen formation was confirmed morphologically by confocal z-stack imaging (Figure 3C), but demonstrating interconnected, perfusable lumens would require additional functional assays.

In this study, constitutive fluorescent labeling was applied only to GFP-expressing HUVECs. For hiPSC-derived cardiomyocytes and for HUVECs that are not GFP-labeled, live-cell dyes can be used to enable fluorescence-based tracking, although dye toxicity and signal stability should be taken into consideration. Using separately tagged iPSC-derived lines with distinct reporters for endothelial cells and cardiomyocytes would provide robust, cell-type-specific visualization throughout the culture period.

Endothelial-to-mesenchymal transition (EndoMT) may occur in compact co-culture environments and could alter endothelial identity over time. Although the sustained stability of vascular networks in our system argues against overt EndoMT, subtle transcriptional shifts cannot be excluded without transcriptomic profiling, which would be a valuable addition in future studies.

Chip assembly and tubing connection are labor-intensive, and multiple junctions introduce points where contamination, leakage, or bubble formation may occur. These mechanical issues can lead to uneven flow or sudden flow interruptions, affecting experimental reliability. Despite providing more physiologically relevant unidirectional perfusion than gravity-driven systems, the external flow setup remains sensitive to handling and requires regular inspection.

In the current configuration, perfusion is restricted to the side channels, and the vascular network within the gel is therefore not exposed to well defined shear stress, an important cue for endothelial barrier function and stability.^19^ Although the chip can be configured for gel perfusion, this would require maintaining a controlled pressure differential across the gel and increases the risk of gel disruption, and was therefore not included here.

Finally, long-term co-culture is feasible in static droplet formats but more difficult under continuous perfusion in the chip. In perfused setups, bubble accumulation is a key limitation and may require additional flow-stabilizing components to support extended culture durations.

## Troubleshooting

### Problem 1

Leaks: Related to Step 15.

Leaks usually arise from insufficiently tightened tubing connections or movement of the chip during handling.

### Potential solution

Ensure that all tubing, connectors, and screws are firmly tightened at Step 15. Minimize repositioning of the chip between the incubator and the microscope. If the chip must be moved, fixate the tubing to a holder using Scotch tape to minimize movement-induced strain on the connectors. This prevents small positional shifts that can lead to leakage.

### Problem 2

Air bubbles in the channels: Related to Step 15.

Air bubbles can form when dissolved gases are released as the cold medium warms to 37°C in the incubator, or when air is introduced during tubing changes or through blind spaces in connectors. Small bubbles usually do not interfere with culture, but channel blockage disrupts medium flow and can compromise cell viability. Preventing bubble entry is therefore essential.

### Potential solution

• Allow the medium to warm to 37°C before loading and avoid air pockets when connecting or disconnecting tubing. Degassing the medium beforehand reduces the likelihood of bubble formation. Any standard degassing method available in the lab is suitable. Alternatively, placing the medium in a syringe with the outlet sealed and gently pulling the plunger can provide partial degassing.
• A bubble trap is an effective additional safeguard. Position the bubble trap as close to the chip as possible so that upstream tubing can be disconnected or reconnected to refresh medium without introducing air into the channels. Although more expensive, bubble traps improve long-term stability and reduce the risk of channel obstruction.

### Problem 3

Cell death inside the chip: Related to Step 15–17.

Cell death often occurs when the medium supply is insufficient. This can happen when the medium in reservoirs is not changed frequently enough, when a small air bubble blocks part of the channel, or when the seeding density is higher than what the medium flow can support.

### Potential solution

First, check that medium delivery is adequate: replace medium at least twice per day when using reservoirs, verify that all channels are perfused, and rule out any trapped bubbles or blockages. To increase nutrient delivery when using reservoirs, place the chips on a gentle rocker.

Only adjust the seeding density once you are sure that medium supply is not the limiting factor. Reducing the density is mainly relevant at high concentrations above roughly 15 x 10^6^ cells per milliliter, and only when perfusion is stable. A flow rate of around 2 microliters per minute can serve as a practical reference, though it is based on experience rather than strict requirements. A continuous, unobstructed medium supply is essential for maintaining cell viability and stable network formation.

### Problem 4

Gel escapes the central channel during loading: Related to Step 12.

Gel escape usually occurs when the pressure applied during loading exceeds the forces that keep the gel confined between the posts. These retaining forces are mainly surface tension and capillary effects created by the geometry of the central lane. When the gel is dispensed too quickly or with too large a volume, the hydrostatic pressure pushes it past the posts into the side channels.

### Potential solution

Use a finer pipette tip or a pipette with a smaller maximum volume. This helps you dispense more slowly and reproducibly, which reduces the pressure peak that can force the gel past the posts. Avoid coating the chip, as coating alters surface tension and reduces the confining effect of the posts, which makes overflow more likely.

### Problem 5

Contamination during chip assembly or culture: Related to Step 15–17.

Microfluidic chips are challenging to assemble under fully sterile conditions because their small components and tight connections often require direct manual handling. Additionally, in most laboratories, the incubator is located outside the hood, so final steps such as connecting tubing to the perfusion pump must be performed in a less controlled environment. These constraints increase the risk of introducing contaminants during setup or transfer.

### Potential solution

Assemble the chip in a flow hood whenever possible and use sterile tweezers or forceps to handle small parts. If direct manipulation is required, disinfect gloves immediately beforehand and avoid contact with non-sterile surfaces. Keep tubing, connectors, and all assembly tools sterile, and maintain a clean environment during transport and incubation. Place each chip in an individual 10- or 15-cm sterile dish to create a clean, self-contained handling unit and minimize direct handling of the chip during transport and incubation.

Because complete sterility is difficult to guarantee, it is advisable to include antibiotics in the culture medium. In our experience, contamination is uncommon when these precautions are taken.

## Resource availability

### Lead contact

Further information and requests for resources and reagents should be directed to and will be fulfilled by the lead contact, Frank G. van Steenbeek (f.g.vansteenbeek@uu.nl).

### Technical contact

Technical questions on executing this protocol should be directed to and will be answered by the technical contact, Frank G. van Steenbeek (f.g.vansteenbeek@uu.nl).

### Materials availability

No new materials have been generated by this study.

### Data and code availability

Datasets and code are available upon request.

## Acknowledgments

The DCVA DOUBLE DOSE grant, Stichting Jubileumfonds and the Maria Naundorf van Gorkum fund supported this work. We gratefully acknowledge Christian van Dijk for providing the GFP-labelled HUVECs. We thank Jolanda van der Velden, Remco Hoogervorst, and Floor den Dolder for generously sharing their expertise and training on hiPSC-derived cardiomyocytes. We acknowledge the support of the Regenerative Medicine Center Utrecht and the Center of Cell Imaging (10.13039/100013504CCI), Utrecht, whose core facilities were essential for this work. We also thank the Medical Technology department in the Utrecht University Medical Center, particularly Pieter Feddema and Fico Ertrop, for facilitating and configuring the perfusion pump used in this study. Figures were created using Biorender.com.

## Author contributions

F.G.v.S. conceived the project and developed the overall experimental strategy. T.C.F.S. performed the key experimental work, including cell culture, handling of hiPSC-derived cardiomyocytes, imaging, data acquisition, analysis, and visualization. A.K. contributed to the optimization of selected experimental steps and supported protocol refinement. C.G.M.v.D. provided the GFP-labelled HUVECs, and J.v.d.V. provided the iPSC-derived cardiomyocytes. R.W. offered technical support, assisting with assay setup, troubleshooting, and the operation of equipment. T.C.F.S. wrote the manuscript. P.v.d.H., M.H., R.J.A.V., F.G.v.S., and J.v.d.V. supervised the project and contributed to the interpretation of the findings. M.H. and J.v.d.V. secured the DCVA DOUBLE DOSE grant that supported this work. All authors reviewed and approved the final manuscript.

## Declaration of interests

The authors declare no competing interests.

## Declaration of generative AI and AI-assisted technologies in the writing process

During the preparation of this work the authors used an AI-based language model (ChatGPT, OpenAI) in order to assist with language editing. After using this tool, the authors reviewed and edited the content as needed and take full responsibility for the content of the published article.

## Supplemental Information

Table S1. Cell suspension and hydrogel loading calculations, related to Step 5 This calculation sheet is used to determine cell numbers, suspension volumes, and reagent amounts required for hydrogel loading in microfluidic chips. The sheet calculates final endothelial cell and hiPSC-cardiomyocyte concentrations in the hydrogel based on user-defined target concentrations, channel volume, and number of lanes. It further provides stepwise volumes for preparing the cell suspension, centrifugation, resuspension, and subsequent 1:1 mixing (v/v) with fibrinogen before gel loading. Users should only modify the input fields indicated in the shaded cells. All other fields contain formulas and should not be edited. Calculations assume a 1:1 volumetric mixing of cell suspension with fibrinogen. A 10% excess volume is automatically included to account for pipetting losses. This calculation sheet is used in Step 5 (Prepare fibrin co-culture suspension and load gels) of the protocol.

Table S2. AngioTool 2.0 parameter settings used for endothelial network quantification; related to Expected Outcomes Overview of the AngioTool 2.0 analysis settings applied for segmentation and quantification of fluorescent endothelial network images, including parameters for vessel detection, skeletonization, and network feature extraction. These settings were kept consistent across all analyzed images to enable reproducible comparison of vascular network morphology.

Table S3. MUSCLEMOTION parameter settings used for cardiomyocyte transient analysis; related to Expected Outcomes Overview of the MUSCLEMOTION analysis settings applied for the analysis of cardiomyocyte contraction transients. MUSCLEMOTION was run in batch mode with automatic reference frame detection and automatic transient analysis enabled.
